# Protein structural changes on a CubeSat under rocket acceleration profile

**DOI:** 10.1038/s41526-020-0102-3

**Published:** 2020-04-23

**Authors:** Autumn Luna, Jacob Meisel, Kaitlin Hsu, Silvia Russi, Daniel Fernandez

**Affiliations:** 1 Mechanical Engineering Department, School of Engineering, Stanford University, Stanford, CA 94305 USA; 2 Electrical Engineering Department, School of Engineering, Stanford University, Stanford, CA 94305 USA; 3 Biology Department, School of Humanities and Sciences, Stanford University, Stanford, CA 94305 USA; 4 Stanford Synchrotron Radiation Lightsource (SSRL), SLAC National Accelerator Laboratories, Menlo Park, CA 94025 USA; 5 Stanford ChEM-H Macromolecular Structure Knowledge Center (MSKC), Stanford University, Stanford, CA 94305 USA; 6 Stanford ChEM-H Institute, Stanford University, Stanford, CA 94305 USA

**Keywords:** Technology, Nanocrystallography, Aerospace engineering

## Abstract

Catalyzing life-sustaining reactions, proteins are composed by 20 different amino acids that fold into a compact yet flexible three-dimensional architecture, which dictates what their function(s) might be. Determining the spatial arrangement of the atoms, the protein’s 3D structure, enables key advances in fundamental and applied research. Protein crystallization is a powerful technique to achieve this. Unlike Earth’s crystallization experiments, biomolecular crystallization in space in the absence of gravitational force is actively sought to improve crystal growth techniques. However, the effects of changing gravitational vectors on a protein solution reaching supersaturation remain largely unknown. Here, we have developed a low-cost crystallization cell within a CubeSat payload module to exploit the unique experimental conditions set aboard a sounding rocket. We designed a biaxial gimbal to house the crystallization experiments and take measurements on the protein solution in-flight with a spectrophotometry system. After flight, we used X-ray diffraction analysis to determine that flown protein has a structural rearrangement marked by loss of the protein’s water and sodium in a manner that differs from crystals grown on the ground. We finally show that our gimbal payload module design is a portable experimental setup to take laboratory research investigations into exploratory space flights.

## Introduction

Second to water, a typical human cell is by mass 20% protein of which there are ~10^4^–10^5^ different species^1^. Proteins sustain the complex life processes of gravity-evolved multicellular organisms and, to a bigger or lesser degree, they can acclimate to changes in gravity as several studies have suggested^2–5^. Reduced gravity offers boundless opportunities for technological exploration in areas such as tissue engineering^6^, biomaterials^7^, energy production^8^, geological prospection^9^, or for in-space supply manufacturing^10^. A further technological application, crystallization of mineral^11^, inorganic^12^, colloidal^13–15^, and macromolecular samples, has been widely explored.

The goal of macromolecular crystallography is to reveal the macromolecule’s three-dimensional (3D) structure at the atom’s level by using X-ray diffraction on crystals obtained from a variety of experimental setups^16^. Crystallization in space begun ~40 years ago with the idea that in microgravity the crystals will grow with enhanced properties that would eventually improve the quality of the derived X-ray diffraction data^17,18^. Yet, protein crystallization in space is not entirely a perturbation-free process as transient accelerations that may affect the crystal growth process originate from onboard human activity and/or space mission operations ^19,20^.

## Results

### In-solution protein experimental cell design

**Fig. 1:**
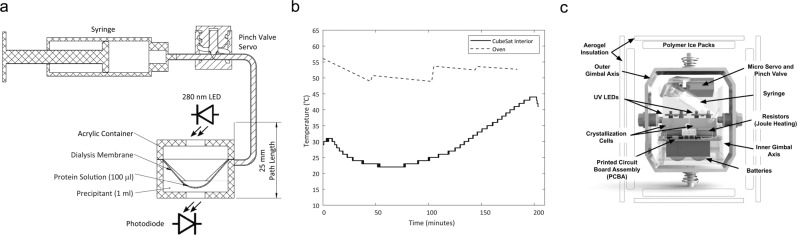
Flowchart for the design of the experimental crystallization cell payload. **a** Schematic view of the payload crystallization cell principle of operation. The crystallization cell is a 25 mm × 25 mm × 19 mm acrylic box that holds the semipermeable cellulose membrane molded into a conical shape to contain the protein and provide a large contact area to the precipitant solution. The precipitant solution, a mixture of buffer, salt, and the water-soluble polymer PEG, is delivered via the syringe-pump device upon launch to initiate crystallization. A low-cost, single-wavelength UV LED and photodiode was coupled to the experiment to monitor the protein solution. **b** Testing insulation at simulating high-temperature launch conditions in the laboratory. The CubeSat unit was assembled with ice packs added in the bulkheads and the unit placed in an oven at 50 °C. Inside the unit, temperature reached a low of 22 °C after adding the ice packs, and then it gradually began to rise at a rate of 0.23 °C/min. Due to the temperature never reaching lower than 22 °C, resistive heating was not employed for the launch. **c** Gimbal interior showing two crystallization cells, deployment syringe, and wiring. An inertial measurement unit was used to measure the orientation and acceleration experienced within the inner gimbal box. A microSD to record experimental data and a printed circuit board assembly around a SAMD21 microcontroller complete the electronics system. To power the system, we used 1100 mAh LiPo batteries and a 3.3 V buck-boost voltage converter.

We sought to understand what these changing acceleration vectors might cause on a supersaturated protein solution when a space mission begins, upon the rocket’s motor burn, and during spaceflight. To this end, we designed a new biaxial gimbal experimental crystallization setup for a 1.5 U CubeSat payload system. First, we built a crystallization cell system based on the well-known bulk dialysis crystallization method employed by the pioneers of protein crystallography^21^. In a diffusion cell, a concentrated solution of protein is separated by a semipermeable membrane from a larger volume of a concentrated solution of precipitant. Diffusion will took place through the membrane retaining molecular species of higher molecular weight with respect to the membrane’s pore size. We used inexpensive acrylic boxes with lid to create the diffusion cell, and to monitor the protein solution, we adapted a circuit with a UV-light-emitting diode and a detector (Fig. 1a). This design allows for a space-efficient, portable configuration, and a cost-effective means to perform crystallization experiments. Although crystal growth rate is marginally important in standard crystallography analysis, it is however critical for our payload experiment. We chose lysozyme as the subject of our study because of its biological relevance and its widespread use in a laboratory setting. Lysozyme belongs to a major family of enzymes that specifically hydrolyze murein, a peptidoglycan which is the insoluble polymer that forms the cell wall of bacteria; in fact, in humans, lysozyme is distributed mostly in tissues in contact to airborne pathogens, suggesting a protective role^22,23^. Adapting a fast crystal-growing procedure^24^, we obtained a large number of crystals within the very limited time window determined by the flight (~60 s).

### Temperature control, gimbal assembly, and recovery of the experimental cell

Then, to successfully recover the protein samples for after-flight analysis, we designed a temperature-controlled (temperature is a critical variable for crystal growth) and shock-reducing (to prevent crystal damage) portable container. On launch ground, temperatures can reach more than 35 °C and they may descend to −45 °C at altitude 9100 m. To keep temperature close to the range crystals were obtained in the preliminary laboratory tests, we resorted to energy-saving, low-weight-practicable resources. Temperature was moderated by polymer ice packs and aerogel insulation encasing the CubeSat structure, and by placing 12 Ω resistors adjacent to the crystallization cell for Joule heating. We conducted laboratory tests with the assembled payload at a temperature close to what was to be expected on the desert launch ground and found that the insulation lining prevented rapid shifts in the inside of the module despite external temperature reaching 50 °C simulated in an oven, even after many hours of high-temperature exposure (Fig. 1b). For temperatures under 20 °C we adopted resistive heating; however, as our flight conditions were towards higher temperatures, it was not needed. Before rocket’s launch, exterior ice packs were placed around the payload to minimize heating, but due to delays, the interior temperature was around 36 °C at time of launch. The CubeSat was secured within the rocket for the duration of the launch. Rocket recovery is composed of two parachute ejection events to reduce landing speed to 8.8 m/s. A 0.76 m diameter spherical parachute and a 1.62 m diameter toroidal parachute are deployed at maximum altitude and 460 m above the ground, respectively. To minimize vibration, we selected a spring-laden, freely moving gimbal subsystem that levels upon the main system’s movements (Fig. 1c). All the necessary hardware to initiate, monitor, and record the experiment are housed in the gimbal, including: the precipitant deployment system, the UV spectrophotometer, the temperature control, the accelerometer, the data acquisition card, electronics, and power supply. The entire gimbal system fits within a 1.5 U CubeSat frame (Supplementary Figs. 1 and 2), is modular and easy-to-integrate into the rocket, and it does not interfere with launch operations.

### In-flight protein solution transformations

**Fig. 2:**
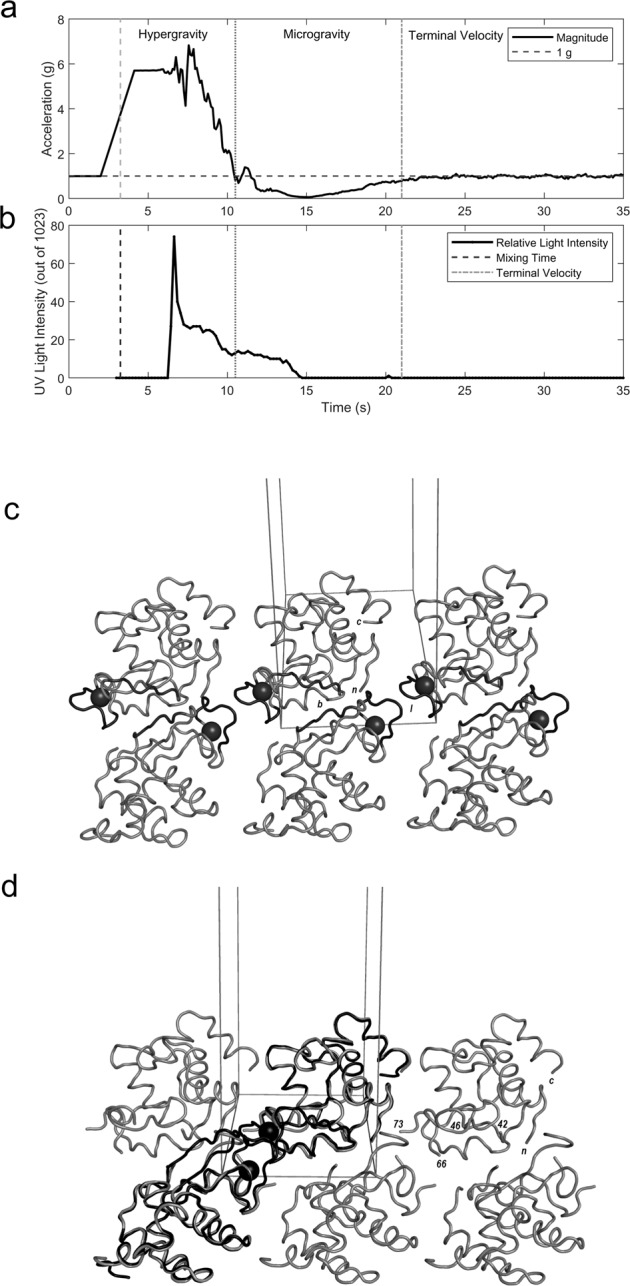
Lysozyme solution evolving to crystals and protein structural changes. **a** Zoom in on flight accelerometer data, and **b** protein solution UV-light measurements. On rocket’s ascent above the 2.9 g preset acceleration threshold, the precipitant solution is injected into the crystallization cell triggering changes to the protein solution on the other side of the semipermeable membrane. The system is firstly under varying acceleration conditions, then the acceleration decreases sharply to microgravity to finally reach terminal velocity (1 g). **c** Protein crystal packing environment of lysozyme in the Earth-grown crystal. Six symmetry-related molecules are depicted along the crystallographic *c* axis. Only backbone atoms from N-terminal Lys1 (labeled *n*) to C-terminal Leu129 (*c*) are shown as a tube in gray. Interface interactions of the β-sheet 43–46 are in dark gray (*b*) and of the sodium-bound loop 61–78 (*l*, in black) are highlighted. The sodium ion (sphere in black) is separated by 14.8 Å from a neighboring molecule ion and the interstices are filled by water molecules (not shown for clarity). **d** Packing of the space-grown lysozyme crystal. Lysozyme backbone atoms from N-terminal Lys1 (*n*) to C-terminal Cys127 (*c*) are shown as a tube in gray. Approximately the same orientation as before. Numbers indicate the positions of amino acids for which coordinates could accurately be determined (positions that could not be traced in the electron density maps include 43–45 of the β-sheet and 67–72 in the long loop 61–78). Two Earth-grown lysozyme molecules (black) were overlaid onto two neighboring space-grown molecules to illustrate that sodium-binding is noncompatible with this dry packing arrangement.

The gimbal system was integrated and assembled into the CubeSat module and launched. With the protein already loaded onto the membrane, the precipitant solution was injected upon rocket’s ascent. To track the evolution of the protein solution while in flight, we used a light-emitting source at 280 nm that traverses the crystallization cell. During rocket’s preparation and gimbal integration the readings of UV-light intensity transmitted through the protein solution were unchanged, but upon motor burn and injection of the precipitant, the solution was under large acceleration during 6.5 s (Fig. 2a, “Hypergravity”). The sharpest spike of transmitted UV intensity is observed at the largest excursion of the acceleration vectors whose overall magnitude reaches 6.82 g (Fig. 2b). After motor burn, the rocket decelerated from drag for 7.5 s, which generated an apparent freefall for the crystallization cell (Fig. 2a, “Microgravity”). During this time, the UV readings show a rapid decrease in intensity, suggesting that the solution transitions to microgravity conditions. After the rocket reached peak altitude, a parachute recovery system was ejected, and the assembly quickly reached terminal velocity (Fig. 2a, “Terminal Velocity”). In this final stage, the crystallization cell experienced 1 g for 42 s and showed non-varying UV readings (Fig. 2b, right side). In summary, the flight accelerometer and UV-light readings indicate marked changes of the directional acceleration magnitudes on the lysozyme solution as it supersaturated with the precipitant to rapidly form nascent microcrystals. However, it was not possible establishing whether a given acceleration vector component acted preferentially on any particular crystal growth dimension, but rather they have altered the crystal properties altogether.

### Protein polymorphism at the atom’s level

To better understand the effect of the rocket’s acceleration profile on the dialysis crystallization experiment, we analyzed an in-flight grown crystal and a ground control crystal through X-ray diffraction. Table 1 lists the crystallographic data. Unexpectedly, the space-grown and Earth-grown crystals are non-isomorphic: the lengths of the crystal lattice vectors in the μg lysozyme collapse by ~6% while the crystal’s solvent content is ~10% less than the 1 g lysozyme. A survey of publicly available data indicates that our μg lysozyme is the smallest among 622 tetragonal lysozyme structures. These results are not inconsistent with previous observations on the dynamic structural flexibility of a related phage lysozyme crystallized under a variety of chemical precipitants^25^, and imply that the protein has replaced water-mediated contacts by protein–protein contacts and/or that changed global conformation. Inspection of the 3D structure reveals that despite their different growth regimes the overall structure is preserved and is indistinguishable with the canonical lysozyme fold. However, the largest positional changes concentrated on two areas that mediate interface contacts with crystal lattice neighbors: at the β-sheet (residues 42–46) and within the long-coiled loop region (residues 66–73). Interestingly, the sodium-binding site within the coiled loop is well organized in 1 g lysozyme (Fig. 2c) while it is abrogated in the μg crystal (Fig. 2d).This finding is in line with a recent study on orthorhombic lysozyme that shows this sodium-binding site is likely to have a conformational change with loss of the metal ion^26^. In 1 g lysozyme, the sodium site is at 14.8 Å of a symmetry-related Na^+^ ion organizing an intermolecular interface thanks to surrounding water. Such an arrangement however cannot be possible made in the μg lysozyme as the smaller lattice vectors would place the Na^+^–Na^+^ sites closer by 1 Å provoking spatial clashes between amino acid side chains (compare Fig. 2c, d). Similarly, the β-sheet changes in the μg crystal to make interfacial contacts to neighbor molecules on a manner that differs from the well organized 1 g lysozyme. Taken together, these results demonstrate that local changes affecting a small portion (10% of the total amino acid components) in flexible, solvent-exposed areas that mediate intermolecular contacts could have made possible the smaller μg crystal without paying the penalty of de-folding lysozyme.

**Table 1:** Data collection and refinement statistics.

	μg Lysozyme	1 g Lysozyme
Data collection		
Beamline	SSRL BL12–2	SSRL BL14-1
Wavelength (Å)	0.97946	1.19499
Space group	P4_3_ 2_1_ 2	P4_3_ 2_1_ 2
Cell dimensions		
* a*, *b*, *c* (Å)	75.97, 75.97, 34.87	78.87, 78.87, 37.15
α, β, γ (°)	90.00, 90.00, 90.00	90.00, 90.00, 90.00
Unit cell/asymmetric unit vol (Å^3^)	201,250/25,156	231,098/28,886
Mosaicity (°)^a^	0.29	0.15
Wilson B-factor^b^	59.6	20.7
Matthews coefficient (Å^3^/Da)^c^	1.73	1.99
Solvent content (%)^c^	29.1	38.3
Resolution (Å)^d^	53.72 (2.67)	39.43 (1.60)
* R*_merge_^e^	0.085 (0.777)	0.045 (0.619)
* I*/σ*I* ratio^f^	11.7 (2.3)	10.9 (1.7)
Completeness (%)^g^	99.6 (99.7)	96.2 (99.6)
Redundancy^h^	6.1 (6.7)	3.2 (3.0)
Refinement		
Resolution (Å)	30.0–2.67	30.0–1.60
No. reflections/test set	2912/209	14,505/782
* R*_work_/*R*_free_^i^	24.4/30.6	17.0/22.0
* F*_obs_ − *F*_calc_ correlation^j^	0.93	0.97
No. atoms		
Protein	874	1014
Ligand/ion	1 (chlorine)	5 (1 sodium, 4 chlorine)
Water	6	140
*B*-factors		
Protein	76.5	24.8
Ligand/ion	65.6	30.1
Water	52.9	38.8
R.m.s. deviations		
Bond lengths (Å)	0.011	0.020
Bond angles (°)	1.44	1.94
Ramachandran statistics^k^		
Most favored regions (%)	100	100
Disallowed regions (%)	0	0

^a^Degree of crystal imperfection, a higher mosaicity contributes to broader (less sharply defined) diffraction intensity profiles.

^b^Overall B-factor value, an approximation to the falloff of atomic scattering with resolution.

^c^Ratio of the volume of the asymmetric unit to the molecular weight of all protein molecules in the asymmetric unit.

^d^Value in parentheses is for the highest-resolution shell: 2.67–2.82 Å in μg lysozyme and 1.60–1.64 Å in 1 g lysozyme.

^e^Reliability factor for symmetry-related reflections calculated as: *R*_merge_ = Σ_hkl_ Σj = 1 to N|I_hkl_ − I_hkl_ (j)|/Σ_hkl_ Σj = 1 to N I_hkl_ (j), where N is the redundancy of the data. In parentheses, the cumulative value at the highest-resolution shell.

^f^Ratio of mean intensity to the mean standard deviation of the intensity over the entire resolution range.

^g^Fraction of measured reflections to possible observations at the resolution range.

^h^Number of measurements of individual, symmetry unique reflections.

^i^Average deviation between the observed and calculated structure factors calculated as: *R*_work_ = Σ_hkl_||*F*_obs_| − |*F*_calc_| |/Σ_hkl_|*F*_obs_|, where the *F*_obs_ and *F*_calc_ are the observed and calculated structure factor amplitudes of reflection hkl. *R*_free_ is equal to *R*_factor_ but for a randomly selected 5.0% (6.3% in μg lysozyme) subset of the total reflections that were held aside throughout refinement for cross-validation.

^j^Correlation coefficient between observed and calculated structure factor amplitudes.

^k^According to Procheck for non-proline and non-glycine residues.

These results suggest that upon launch the protein solution experienced a shock such that by hypergravity solutes of lower molecular weight than the cutoff of the semipermeable membrane were passed through to the bulk precipitant solution. We cannot exclude the possibility however that the acceleration vectors acting on the protein molecule might have triggered the conformational change on the flexible, solvent-exposed areas and that the protein remodeled the sodium-binding site, releasing it. Either way, the low-solvent content μg crystal is the smallest among the tetragonal lysozyme crystals with available 3D coordinates. By overall crystallographic statistics our rocket-grown crystal was of lower quality than the ground lysozyme crystal raising the possibility that our design was not a problem-free container for this fragile cargo. This cannot be ascribed only to hardware though as a previous study concluded that dissimilar protein crystallization outcomes aboard a sounding rocket would have been caused by factors related to reentry or by the crystal growth process itself^27^. To summarize, our gimbal design rendered an experimental setup for a biophysical experiment that, shuttled in a CubeSat frame, showed the altering effects of a rocket’s acceleration profile on a protein in solution.

## Discussion

We developed a biaxial system to be fitted into a 1.5 U CubeSat as a biology research experiment payload and adapted a protein crystallization experiment to span the very short timeframe of the rocket’s flight. Crystal growth under rocket’s flight conditions affected the way the protein molecules assembled in the crystal, driving local conformational changes that resulted in the loss of water and protein-bound sodium. The limited timescale of the flight would however render our system of little utility to most protein crystallization experiments, even though for difficult crystallization problems the simplicity of the method of bulk diffusion could be used for screening crystallization conditions on a larger scale. In this work and in previous reports^28,29^ with model proteins, high-g forces produced crystals from or by achieving supersaturated solutions. The goal would be obtaining nuclei or nascent crystals to be used as seeds for conventional crystallization on Earth or under microgravity in the International Space Station. A more suitable recovery system for the CubeSat experimental cell and systematic exploration at high-g on a variety of precipitants and macromolecules would be needed to test the feasibility of the system. Additional application of the experimental cell on a sounding rocket would be in quickly evolving systems such as enzyme kinetics, where these reactions occur typically on the order of milliseconds or less and would require tightly controlled, fast solution-mixing, and quick data acquisition systems. Interestingly, we have demonstrated here that rocket’s acceleration readings triggered a pump-controlled injection of a chemical reactant that ultimately led to protein structural changes. Incorporating spectral monitoring and fast-acting syringe pumps our device would promise an easy-to-integrate, portable, and low-cost platform for pilot studies destined to better understand fundamental biological process in space activities.

## Methods

### Crystallization reagents and apparatus

Commercially available hen egg white lysozyme lyophilized powder was from MP Biomedicals, LLC (Solon, OH, USA). Sodium acetate (Mallinckrodt Baker, Inc., Paris, KY, USA), sodium chloride (Fisher Scientific, Fair Lawn, NJ, USA), polyethylene glycol 6000 (Sigma-Aldrich, Merck KGaA, Darmstadt, Germany) were dissolved in ultrapure Milli-Q water (EMD Millipore, Merck KGaA, Darmstadt, Germany) and the stock solutions filtered through a 0.45-μm syringe filter. Lysozyme working solutions were made by weighing and dissolving the lysozyme in 50 mM sodium acetate buffer, pH 4.5. Protein was dialyzed/concentrated using a 3 kDa MW cutoff microcon centrifugal device (EMD Millipore, Merck KGaA, Darmstadt, Germany). Protein concentration was measured using a NanoDrop 2000c UV–Visible spectrophotometer (Thermo Fisher Scientific, Wilmington, DE, USA). The crystallization cell was a 25 mm × 25 mm × 19 mm acrylic box purchased from a home goods products store. We chose a regenerated cellulose tubing commonly used for dialysis in the laboratory as a readily available, cheap, and biocompatible medium. We utilized a semipermeable cellulose membrane of 7 kDa MW cutoff without any activation as instructed by the manufacturer (Thermo Fisher Scientific, Wilmington, DE, USA). Square pieces of membrane of 50 mm × 50 mm were cut and molded with a machined finger tool before attachment to the container box. The membrane was molded into a conical shape such that the vertex is in contact to the precipitant solution and its external rim provides tightening for the lid sealing the chamber.

### Design of the crystallization cell and gimbal system

We developed our own microfluidic precipitant deployment system, including a syringe-pump and pinch valve, which a micro servo motor releases upon launch. This syringe-pump design delivers the 2 ml of precipitant solution to the parallel crystallization cells in one second. A low-cost, single-wavelength UV LED and photodiode spectrophotometer was coupled to the experiment to measure the protein absorbance at 280 nm. The UV photodiode has a 250–310 nm responsivity band. The spectrophotometer system was tested in the laboratory to determine the gain and absorbance of the components of the crystallization cell. The box material is not UV-light-transparent and cutouts were done on the acrylic box and lid and sealed with polyethylene film to provide the maximal possible intensity excursion. At a distance of 25 mm to the photodiode, the UV absorption intensity of the experimental box was decreased by only 1.35% compared with the intensity measured in its absence. An inertial measurement unit (Bosch Sensortec BNO055) is used to measure the orientation and acceleration experienced within the inner gimbal box. A microSD to record experimental data and a printed circuit board around a SAMD21 microcontroller complete the electronics system. Again, for space constraints in the inner gimbal box, we used 1100 mAh, 3.7 V, 18350 LiPo batteries, and a 3.3 V buck-boost voltage converter. To provide thermal control we chose long-lasting ice packs, aerogel fiber insulation, and Joule heating. In all, the main power consumption is for heating (57%) and to power the UV LED, sensor, microcontroller, accelerometer, and SD reader (33%). The remainder 10% is left as safety margin. To start the crystallization experiment, we use linear acceleration data to detect launch and to prevent false-starts from jostling the system during integration, an acceleration threshold of 2.9 g for 250 ms was selected.

### Crystal handling, data collection, and structure determination

Crystals from recovered crystallization boxes were harvested directly from the dialysis membrane using synthetic fiber cryo-loops imbibed in Paraton-N oil (Hampton Research, Aliso Viejo, CA, USA). The crystals were immediately plunged into liquid N_2_ for storage for data collection at the synchrotron. Data for μg and 1 g lysozyme were collected at SSRL^30^ beam lines 12-2 and 14-1, respectively. Crystallographic data, refinement statistics and validation reports are presented in Table 1. The X-ray diffracting power of space-grown and Earth-grown crystals differed qualitatively. The highest dispersion for a space-grown crystal was to a minimum Bragg spacing of 2.67 Å. The crystal belonged to the tetragonal space group P4_3_ 2_1_ 2, with unit cell dimensions: *a* = *b* 75.97 Å, *c* = 34.87 Å, *α* = *β* = *γ* = 90°, and contained one polypeptide chain per asymmetry unit. Earth-grown crystals of unit cell dimensions: a = *b* 78.86 Å, *c* = 37.15 Å, *α* = *β* = *γ* = 90°, belonged to the same space group but showed comparatively better X-ray diffraction power to a minimum Bragg spacing of 1.6 Å. The structure of the 1 g lysozyme was solved by the molecular replacement method with EPMR^31^ using the polypeptide chain of PDB: 5KXK^32^ as the search model. Residues 1–129 were unambiguously traced in the electron density map of the 1 g lysozyme, which was then used to phase the μg crystal data with Phaser^33^. The latter could be traced for the polypeptide length except for regions 43–45, 67–72, and 128–129 for which very weak electron density prevented defining an accurate model. In the μg crystal, residues 41–51 deviate on average 1.5 Å from the 1 g lysozyme, which prevented defining an accurate modeling of atomic positions in the μg crystal for residues 43–45. No electron density exists for a metal ion, and, in fact, very weak and poorly defined electron density is evident for the nearby atoms (residues 67–72 could not be located and were not modeled). Refinement rounds using REFMAC^34^ with electron density map inspection in COOT^35^ were iteratively performed until refinement progressed to convergence. Data were reduced with XDS^36^ or Mosflm^37^ and scaled with SCALA^38^. Throughout the structure determination and analysis, software within the CCP4 suite was used^39^. Data validation against reference crystallographic data was done with Procheck^40^. Graphic renderings were performed with Pymol ^41^.

The 1 g lysozyme unit cell vectors fall well within the range found among tetragonal lysozyme crystal structures available from the Protein Data Bank^42^ (average value over 622 structures, SD in parentheses): *a* = *b* = 78.59 (0.84) Å, *c* = 37.54 (0.62) Å; range *a* = *b* = 75.89–81.41 Å, *c* = 35.36–39.96 Å whereas the μg lysozyme lattice vectors are the shortest.

## Supplementary information

supplementary-materials

## Data Availability

Structural data that support the findings of this study have been validated against reference standards and made publicly available within the Protein Data Bank with accession codes 6W7P (1 g lysozyme) and 6W8E (μg lysozyme).
