# Supplementary material for: Protein structural changes on a CubeSat under rocket acceleration profile
Source: NPJ Microgravity. 2020 Apr 23;6:12. doi: 10.1038/s41526-020-0102-3 (PMC7181844; doi:10.1038/s41526-020-0102-3)
Supplement: Supplementary file 1 — supplementary-materials [file 41526_2020_102_MOESM1_ESM.pdf]

Supplementary Figures

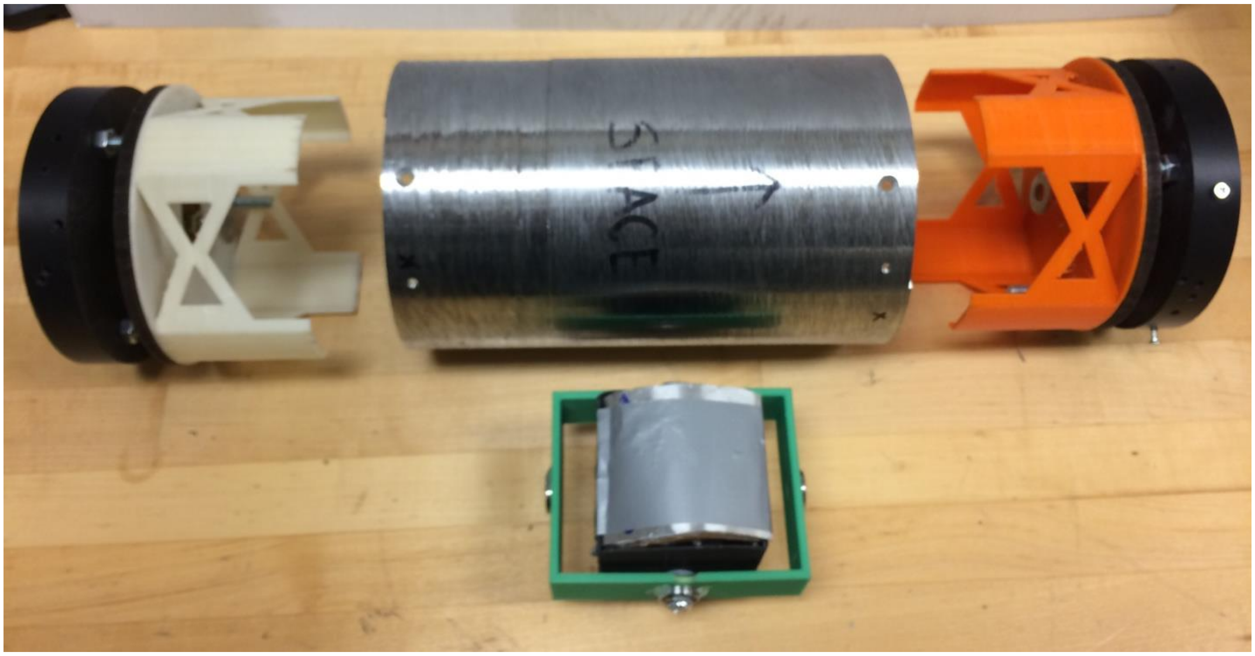

Image 1: Deconstructed assembly of CubeSat frame within rocket body. Polymer ice packs and aerogel insulation sheets are to be lined in the bulkhead's volume between the CubeSat and steel cylinder. The gimbal system (green box) is shown assembled underneath the CubeSat.

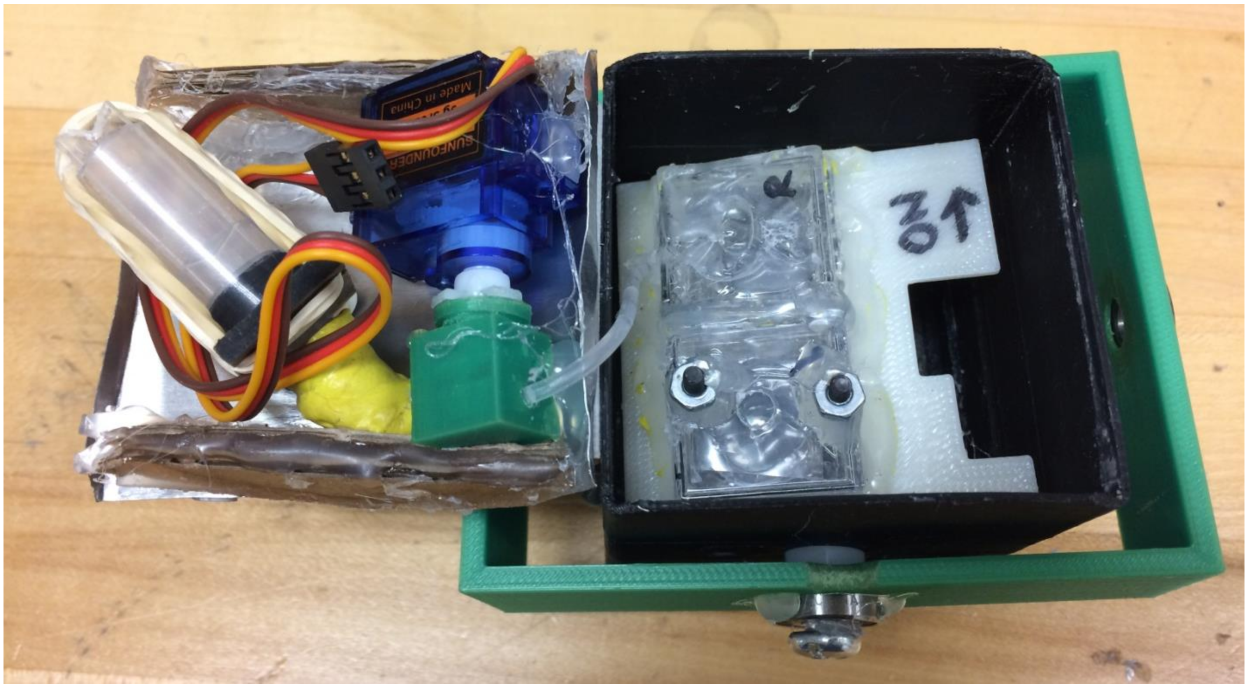

Image 2: Assembled inner gimbal containing crystallization cells (right), micro servo motor, pinch valve, and syringe pump (lid of the box). The circuit board lies underneath the experimental cells.
